# Integrated Multi-omics Analyses Identify CDCA5 as a Novel Biomarker Associated with Alternative Splicing, Tumor Microenvironment, and Cell Proliferation in Colon Cancer Via Pan-cancer Analysis

**DOI:** 10.7150/jca.91082

**Published:** 2024-01-01

**Authors:** Xinyue Bao, Xin Leng, Tianyu Yu, Junzheya Zhu, Yunhan Zhao, Tenzindrogar, Zhiluo Yang, Shaobo Wu, Qi Sun

**Affiliations:** 1 Department of General Surgery, The First Affiliated Hospital of Xi'an Jiaotong University, Xi'an 710061, China.; 2 Department of Urology, Affiliated Kunshan Hospital of Jiangsu University, Suzhou215300, China.

**Keywords:** CDCA5, pan-cancer analysis, biomarker, prognosis, TCGA

## Abstract

**Background**: CDCA5 has been reported as a gene involved in the cell cycle, however current research provides little details. Our goal was to figure out its functions and probable mechanisms in pan-cancer.

**Methods**: Pan-cancer bulk sequencing data and web-based analysis tools were applied to analyze CDCA5's correlations with the gene expression, clinical prognosis, genetic alterations, promoter methylation, alternative splicing, immune checkpoints, tumor microenvironment and enrichment. Real‑time PCR, cell clone formation assay, CCK-8 assay, cell proliferation assay, migration assay, invasion assay and apoptosis assay were used to evaluate the effect of CDCA5 silencing on colon cancer cell lines.

**Results**: CDCA5 is highly expressed in most tumors, which has been linked to a poor prognosis. Immune checkpoints analysis revealed that CDCA5 was associated with the immune gene CD276 in various tumors. Single-cell analysis showed that CDCA5 correlated with proliferating T cell infiltration in COAD. Enrichment analysis demonstrated that CDCA5 may modify cell cycle genes to influence p53 signaling. The examination of DLD1 cells revealed that CDCA5 increased the proliferation and blocked cell apoptosis.

**Conclusion**: This study contributes to the knowledge of the role of CDCA5 in carcinogenesis, highlighting the prognostic potential and carcinogenic involvement of CDCA5 in pan-cancer.

## 1. Introduction

Globally, cancer is the most severe public health problem and the primary cause of death 1. Cancer bioinformatics, which integrates cancer knowledge with information technology, can be a beneficial instrument for advancing cancer diagnosis, prognosis, and therapy 2. Due to the intricacy of the cancer initiation mechanism, pan-cancer analysis is essential for identifying the carcinogenic principle. As a consequence of the publicly-supported TCGA project and the openly accessible GEO database, functional genomic studies can be conducted on all forms of cancer 3.

The CDCA5 protein encoded by the cell division cycle-associated 5 (CDCA5) gene is essential for sister chromatid cohesion as well as segregation 4 . CDCA5 maintains the cohesive complex, preserving sister chromatid cohesion and ensuring accurate chromosome segregation during mitosis and meiosis; it is also required for DNA repair 5. In addition, CDCA5 affects the activity of transcription factors and proteins associated with the cell cycle; it thus promotes cancer cell growth and participation in apoptosis 6. Moreover, studies reveal that CDCA5 serves an important part in the progression of a wide range of malignancies, including bladder cancer, prostate cancer, ovarian cancer and breast cancer, and participates in a variety of signaling pathways, such as the ERK signaling pathway and the PI3k/AKT/mTOR pathway 7-10.

Increasing numbers of research projects are undertaken on CDCA5 each year. However, a comprehensive bioinformatics investigation of the activities of CDCA5 in carcinogenesis has not yet been conducted. We conducted the first pan-cancer analysis of CDCA5 using data from the TCGA project and the GEO database. In our work, we analyzed CDCA5 expression, survival status, DNA promoter methylation, mutations in genes, alternative splicing, immunological infiltration, tumor immune single cells, and enrichment. To investigate the effect of CDCA5 silencing on colon cancer cell lines, cell experiments were conducted. We evaluate all of these factors to investigate the potential molecular mechanism of CDCA5 in the progression or clinical prognosis for various cancers.

## 2. Materials and methods

### 2.1. Gene expression analysis of CDCA5

TIMER2 (Tumor immune estimation resource, version 2, http://timer.cistrome.org/) web is used to observe the differential expression of CDCA5 in different cancer types and normal matched tissues, which were retrieved from TCGA project. For certain malignancies devoid of normal or with extremely limited normal tissues, GEPIA2 (Gene expression profiling interactive analysis, version 2, http://gepia2.cancer-pku.cn/#analysis) web server is used to paint box plots of the expression difference between these tumor tissues and the corresponding normal tissues of the GTEx (Genotype-tissue expression) database 11. Using the "Pathological Stage Plot" module of GEPIA2, we also obtained violin plots of CDCA5 expression in distinct pathological stages of all TCGA tumors. The log2 [TPM(Transcripts per million) + 1] transformation was applied to the expression data for box and violin plots. It is essential to determine the CDCA5 protein expression in multiple organs. The UALCAN portal (http://ualcan.path.uab.edu/index.html), an interactive web resource for studying cancer omics data, was used to analyze a total expression level of CDCA5 protein and CDCA5 promoter methylation 12-14. The Human Protein Atlas (HPA) database (https://www.proteinatlas.org/) was used to further analyze the expression of CDCA5 protein in several organs.

### 2.2. Survival prognosis analysis

Using the "Survival Map" module of GEPIA2, we obtained overall survival (OS) and disease-free survival (DFS) significance map data for CDCA5 across all TCGA tumors. Expression thresholds of cutoff-high (50%) and cutoff-low (50%) values were used to divide the high-expression and low-expression cohorts. Hypothesis testing was conducted using the log-rank test, and survival plots were generated using the "Survival Analysis" module of GEPIA.

### 2.3. Genetic alteration analysis

cBioPortal webtool (http://cbioportal.org/) was utilized to assess the genetic alteration of CDCA5 15, 16. In the "Cancer Types Summary" module, the results of the alteration frequency, mutation type, and copy number (CNA) across all TCGA tumors were displayed. We also obtained the mutated site information of CDCA5 in the “Mutations” module. Additionally, we used the "Comparison/Survival" module to evaluate the disease-specific, progression-free survival differences for esophageal adenocarcinoma (EAC) with or without CDCA5 genetic alteration. The Kaplan-Meier plotter was used to graphically display the pertinent data, and the log-rank test was applied to determine statistical significance.

### 2.4. Alternative splicing analysis of CDCA5

The OncoSplicing database (http://www.oncosplicing.com) is a database designed to comprehensively examine clinically relevant alternative splicing (AS) 17. We used it to search for the AS events of CDCA5 included in both the SplAdder and the SpliceSeq projects. The PanPlot was displayed to show the percent spliced-in (PSI) of TCGA cancers and GTEx tissues. The PanDiff plots were displayed in order to assess the PSI differences of the AS events (detected in more than three tumors) between malignancies and nearby or GTEx normal tissues. The Kaplan-Meier curves were then utilized to investigate the prognostic importance of the AS events in pan-cancer.

### 2.5. Tumor microenvironment (TME) analysis and immune checkpoint analysis

With R packages, we compared the degree of infiltration of 24 immune cells in various tumors. Tumor Immune Estimation Resource (TIMER, https://cistrome.shinyapps.io/timer/) is a database-driven online tool that computes immune cell infiltration scores for primary immune cell types 18, 19. We utilized it and TIMER2 to find the correlation between CDCA5 expression and the infiltration level with B cells, CD4+ T cells, CD 8+ T cells, macrophages, neutrophils, and dendritic cells.

We employed the "Gene_Corr" module of TIMER2 to analyze the correlation between CDCA5 expression and expression of several immune checkpoint genes across all cancer types.

Tumor Immune Single-Cell Hub (TISCH, http://tisch.comp-genomics.org) offered comprehensive annotations of cell types and enabled interactive single-cell transcriptome visualization 20. Multiple datasets at the single-cell or cluster level were utilized to examine the TME of colon cancer.

### 2.6. Enrichment analysis of CDCA5

The STRING (https://string-db.org/) is a webtool to construct a Homo Sapiens CDCA5 co-expression network 21. We set the following main parameters: minimum required interaction score [“Low confidence (0.150)”], meaning of network edges (“evidence”), max number of interactors to show (“no more than 50 interactors” in 1st shell) and active interaction sources (“experiments”). Using the datasets of all TCGA tumor and normal tissues, the "Similar Gene Detection" module of GEPIA2 was utilized to identify the top 100 CDCA5-correlated targeting genes. Additionally, we utilized the "correlation analysis" module of GEPIA2 to conduct a pairwise gene Pearson correlation study between CDCA5 and chosen genes. Applying the log2 TPM on the dot plot. Indicated were the P-value and the correlation coefficient (R). In addition, we utilized the "Gene_Corr" module of TIMER2 to provide the heatmap data of the chosen genes, which includes the partial correlation (cor) and P-value from the purity-adjusted Spearman's rank correlation test. To compare the genes encoding proteins that bind to CDCA5 with the genes that interact with CDCA5, a Venn diagram was used to identify the genes at the intersection of these two sets. KEGG (Kyoto encyclopedia of genes and genomes) pathway and GO (Gene ontology) enrichment analysis were then performed on the genes encoding CDCA5-binding proteins and the CDCA5-interacting genes using the R, version 4.1.2 22, 23.

### 2.7. Cell culture

The human colorectal cancer cell line DLD1 was purchased from Procell Life Science& Technology Co., Ltd (Wuhan,China), which was cultured in DMEM supplemented with 10% heat‑inactivated fetal bovine serum (FBS) (Sijiqing Biologic, Hangzhou, China) and was incubated at 37˚C in a humid incubator with air containing 5% CO2. CDCA5 siRNA#1 sequences were as follows: sense: 5'-CCUCUUCUUGACCUGAACAAUTT-3'; antisense: 5'-AUUGUUCAGGUCAAGAAGAGGTT-3'. CDCA5 siRNA#2 sequences were as follows: sense: 5'-CCAAAUACUUUCGGACCCAAATT-3'; antisense: 5'-UUUGGGUCCGAAAGUAUUUGGTT-3'.

### 2.8. Quantitative real‑time PCR

RNA was isolated from cells using TRIzol reagent (Solarbio Science and Technology Co. Beijing, China). Quantitative PCR analysis was performed with the SYBR-Green PCR master mix (TransGen Biotech Beijing, China) on a CFX96 Real-Time PCR detection system (Bio-Rad). Primer sequences were as follows: CDCA5 forward, 5'-TGTGCTCCAAACTCACCGAG-3' and reverse 5'-TCATCCAGCTCCGTTTTCAAG-3'. Relative mRNA expression levels were determined with the internal control GAPDH using the 2‑ΔΔCq method.

### 2.9. Cell clone formation assay

After transfection, cells were digested and counted. Each group's cells were seeded into a 6-well plate at a density of 1*10^3^ well. Following 14 days of incubation in a CO2 incubator, the cells were fixed with polyformaldehyde and stained with crystal violet. A limited cluster of over 100 cells is considered a colony. Using ImageJ software, clone formation was photographed and the number of colonies was determined.

### 2.10. CCK-8 assay and cell proliferation assay

After 24 hours of transfection with siRNA of CDCA5, DLD1 cells were seeded into microplates for further detection. Cell viability assays were performed using Cell Counting Kit-8 (CCK8) colorimetric assay (Fude Biological Technology, Hangzhou, China) at a wavelength of 450 nm by spectrophotometry. Cell proliferation was evaluated by 5-ethynyl-2ʹ-deoxyuridine (EdU) staining according to manufacturer' s instructions (Bioscience Biological Technology, Shanghai, China).

### 2.11. Migration assay and invasion assay

Transfected cells were seeded into 8-m pore inserts for transwell migration tests after 24 hours. To induce cell migration, 1% FBS was added to the upper chamber, whereas 10% FBS was added to the lower chamber. After 48 hours of migration, the cells were stained with 0.1% crystal violet and examined using a microscope. The cell invasion tests resembled the assay for cell migration. ImageJ software was utilized to count cells.

### 2.12. Apoptosis assay

According to the instructions of the kit (Bioscience Biological Technology, Shanghai, China), cells were digested with pancreatin without EDTA and collected, recovered in the medium for 30min and then suspended. Annexin V FITC and PI working fluid were added sequentially as specified, and the mixture was incubated on ice against light for 15 minutes. When the cells were resuspended, Cytoflex flow cytometer (Becman,CA, USA) was used to identify apoptosis. The stimulated reception wavelength of FITC-Annexin V is 488/530 mm, whereas the PI emission spectrum is around 617 nm.

### 2.13. Statistical analysis

The SPSS 22.0 program (IBM Company, Armonk, NY, USA) was used to analyze the data. The chi-square and Fisher's exact probability tests were used to examine the count data, while the Student's t-test was used to study the measurement data. The Kaplan-Meier and log-rank tests were used to assess survival curves. Experimental data were presented as mean ± SEM. GraphPad Prism software was used for statistic graphs and statistical analysis. The comparison between two unpaired groups was performed using Student's t-test. p < 0.05 was considered statistically different.

## 3. Results

### 3.1. Gene expression analysis of CDCA5

**Figure 1:**
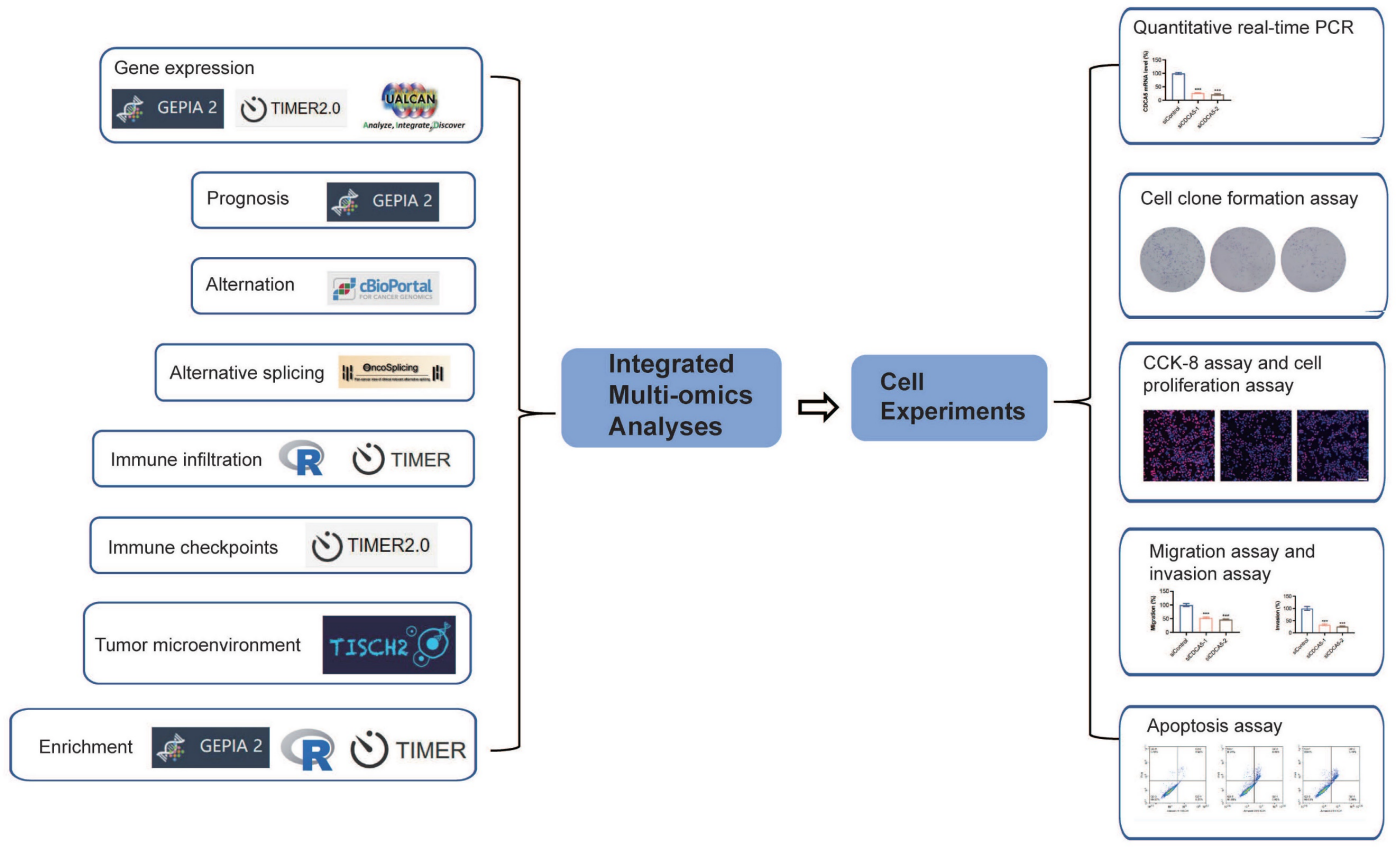
A summary of the entire study. A variety of methods and webtools are utilized in the study.

**Figure 2:**
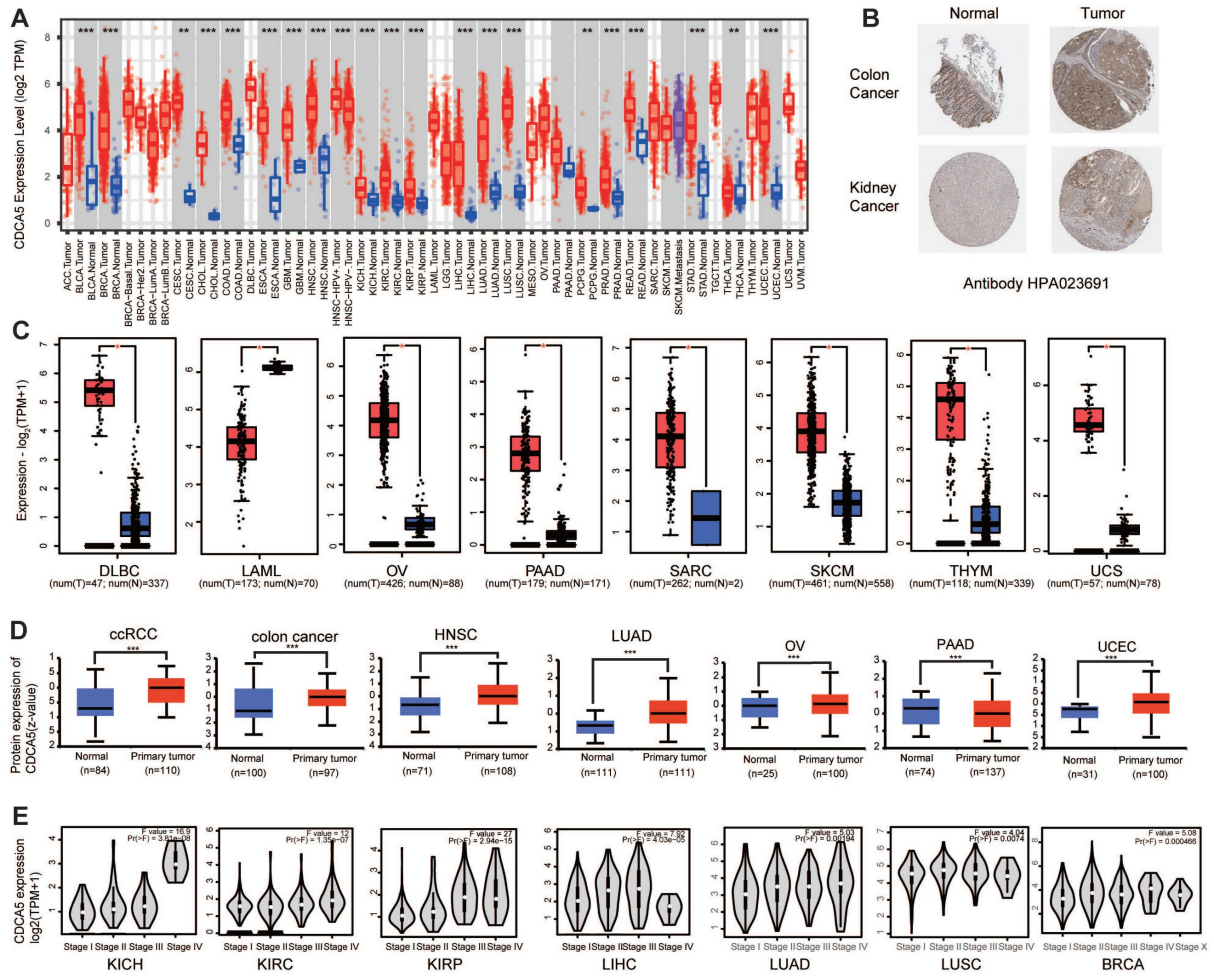
The expression status of CDCA5 gene in various tumors and pathological phases. (A) The mRNA of CDCA5 gene expression levels in different cancers or specific cancer subtypes were analyzed through TIMER2. (B) IHC results obtained from HPA database, show different CDCA5 protein expression between normal tissue and tumor in colon and kidney cancers, using the Antibody HPA023691. (C) As controls for the DLBC, LAML, OV, PAAD, SKCM, THYM, and UCS types in the TCGA project, the matching normal tissues from the GTEx database were included. Box plot data were provided. (D) Utilizing the CPTAC dataset, the expression levels of CDCA5 total protein between normal tissue and primary tissue of clear cell RCC, colon cancer, head and neck squamous carcinoma, lung adenocarcinoma, ovarian cancer, pancreatic adenocarcinoma, and UCEC were compared. (E) The mRNA expression levels of the CDCA5 gene were evaluated by the major pathological phases of KICH, KIRC, KIRP, LIHC, LUAD, LUSC, and BRCA using data from TCGA. Log2 (TPM + 1) was used for log-scale. * P <0.05, ** P <0.01; *** P <0.001.

Figure 1 presents a flowchart illustrating the comprehensive process of this investigation. The expression status of CDCA5 was analyzed across multiple TCGA cancer types utilizing the TIMER2 method. Figure 2A displays that the expression level of CDCA5 in the tumor tissues of Bladder urothelial carcinoma (BLCA), Breast invasive carcinoma (BRCA), Cholangiocarcinoma (CHOL), Colon adenocarcinoma (COAD), Esophageal carcinoma (ESCA), Glioblastoma multiforme (GBM), Head and neck squamous cell carcinoma (HNSC), Kidney chromophobe (KICH), Kidney renal clear cell carcinoma (KIRC), Kidney renal papillary cell carcinoma (KIRP), Liver hepatocellular carcinoma (LIHC), Lung adenocarcinoma (LUAD), Lung squamous cell carcinoma (LUSC), Prostate adenocarcinoma (PRAD), Rectum adenocarcinoma (READ), Stomach adenocarcinoma (STAD), Uterine corpus endometrial carcinoma (UCEC) (P <0.001), Cervical squamous cell carcinoma and endocervical adenocarcinoma (CESC), Pheochromocytoma and paraganglioma (PCPG), and Thyroid carcinoma (THCA) (P <0.01) is higher than the matched control tissues.

Based on the data presented in Figure 2C, normal tissue samples from the GTEx dataset were incorporated as controls, we evaluated the differential expression of CDCA5 between normal and tumor tissues in Lymphoid neoplasm diffuse large B-cell lymphoma (DLBC), Acute myeloid leukemia (LAML), Ovarian serous cystadenocarcinoma (OV), Pancreatic adenocarcinoma (PAAD), Sarcoma (SARC), Skin cutaneous melanoma (SKCM), Thymoma (THYM) and Uterine carcinosarcoma (UCS) with a significance level of P <0.05. As demonstrated in Figure S1A, there was no statistically significant difference between Adrenocortical carcinoma (ACC), Brain low-grade glioma (LGG), and Testicular germ cell tumors (TGCT).

After excluding cancers with a small sample size (Figure S1B), we have observed a significant increase in the total protein expression of CDCA5 in primary tissues of clear cell RCC, colon cancer, head and neck squamous carcinoma, ovarian cancer, lung adenocarcinoma, and UCEC compared to normal tissues based on the CPTAC dataset. However, the total expression of CDCA5 protein in PAAD tissues was significantly lower than that in corresponding normal tissues (Figure 2D; P<0.001). Based on the HPA database, an immunohistochemistry (IHC) analysis was conducted to confirm CDCA5 mRNA expression at the cellular level with corresponding protein expression data (Figure S1C). High levels of cytoplasmic/membranous and nuclear immunoreactivity were detected in colon and kidney cancers, as determined by the HPA023691 antibody (Figure 2B).

Additionally, a clinical correlation analysis was performed to establish the association between CDCA5 mRNA expression and tumor pathological stages. The clinical correlation analysis included various tumor types from TCGA, revealing significant differences in CDCA5 mRNA expression between KICH, KIRC, KIRP, LIHC, LUAD, LUSC, BRCA and their respective normal matched tissues (Figure 2E; P<0.05).

### 3.2. Survival analysis of CDCA5

**Figure 3:**
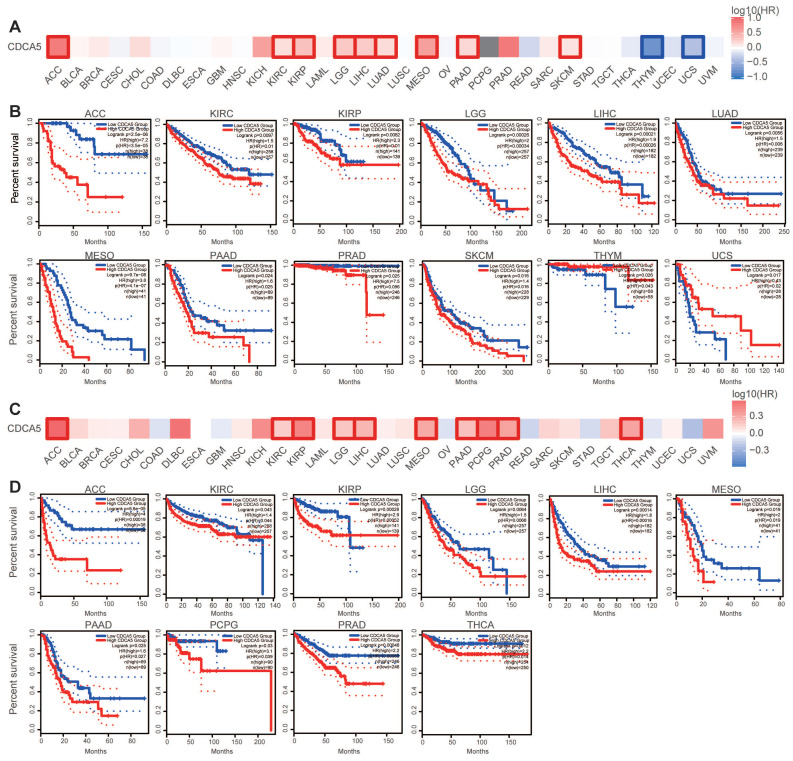
Correlation between CDCA5 gene expression and survival prognosis of cancers in the TCGA database. GEPIA2 was employed to analyze overall survival (A, B) and disease-free survival (C, D) of various tumors in TCGA by CDCA5 gene expression. The survival map and Kaplan-Meier plots with significant results are provided. Red and blue dashed lines represent the 95% confidence intervals for overall survival in the high and low CDCA5 groups, respectively.

We categorized cancer cases into high and low expression groups based on the level of CDCA5 expression, and primarily utilized TCGA and GEO datasets to assess the correlation between CDCA5 expression and patient prognosis across various cancers. Cancers with elevated CDCA5 expression levels are associated with a dismal prognosis for overall survival (OS), such as ACC (P=2.5E-06), KIRC (P=0.0097), KIRP (P=0.0082), LGG (P=0.00026), LIHC (P=0.00021), LUAD (P=0.0056), MESO (P=9.7e-08), PAAD (P=0.024), PRAD (P=0.025), and SKCM (P=0.016) (Figure 3A; Figure 3B). Furthermore, reduced CDCA5 gene expression was linked to unfavorable overall survival prognosis in THYM (P=0.026) and UCS (P=0.024).

Analysis of disease-free survival (DFS) data demonstrated that cancers with high CDCA5 expression have a poor prognosis of ACC (P=6.6e-05), KIRC (P=0.043), KIRP (P=0.00028), LGG (P=0.0064), LIHC (P=0.00014), MESO (P=0.019), PAAD (P=0.025), PCPG (P=0.03), PRAD (P=0.00048), and THCA (P=0.012) (Figure 3C; Figure 3D).

### 3.3. Gene promoter methylation analysis of CDCA5

**Figure 4:**
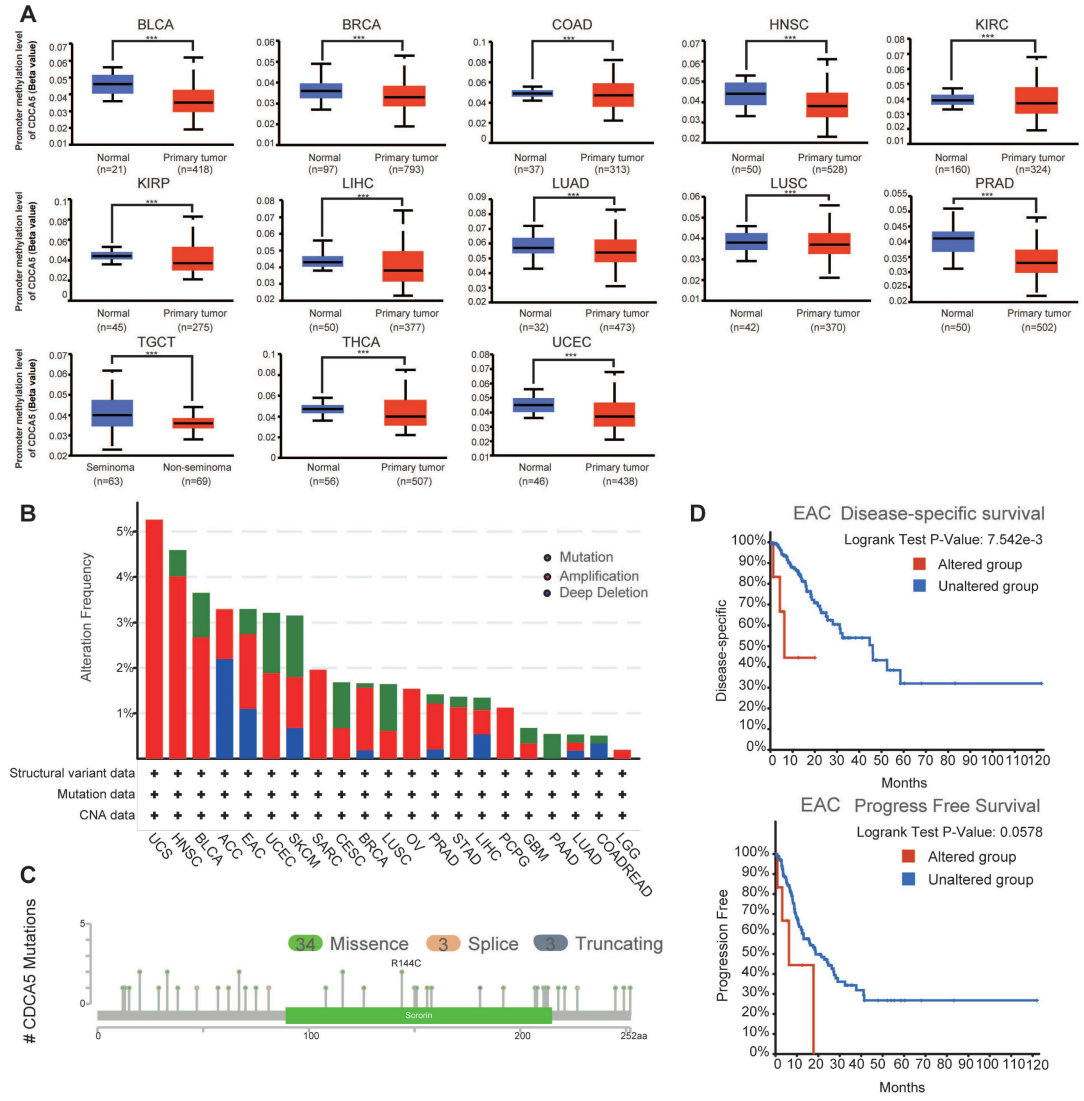
Changes in the CDCA5 DNA molecule in various TCGA cancers. (A) The promoter methylation levels of CDCA5 across 13 types of tumors. *** P <0.001. (B) The alteration frequency with CDCA5 genetic alteration type in different tumors. (C) CDCA5 mutation site. (D) The association between mutation status and disease-specific survival and progression-free survival of EAC.

Using the UALCAN dataset, an analysis of the methylation levels of the CDCA5 gene promoter showed the possible role of CDCA5 across all cancer types. After eliminating tumors with insufficient sample size (Figure S1D), significantly reduced methylation levels of the CDCA5 promoter were seen in 13 kinds of cancer, including BLCA, BRCA, COAD, HNSC, KIRC, KIRP, LIHC, LUAD, LUSC, PRAD, TGCT, THCA, and UCEC (Figure 4A; P<0.001).

### 3.4. Genetic alteration analysis of CDCA5

Subsequently, cBioPortal was utilized to scrutinize the genomic aberrations of CDCA5 across diverse malignancies encompassed in TCGA datasets. Based on the analysis of Figure 4B, individuals with uterine carcinosarcoma (UCS) of the "amplification" subtype exhibit the highest frequency (>5%) of CDCA5 alterations. It is interesting to emphasize that copy number amplification was the predominant form of genomic mutation seen in TCGA tumor samples. Figure 4C depicts a total of 40 CDCA5 alterations, including 34 missense mutations, 3 truncating mutations, and 3 splice mutations (Supplementary Table S1). We subsequently explored the correlation between genetic mutations in CDCA5 and clinical outcomes of cancer patients. EAC patients with altered CDCA5 had a lower prognosis for disease-specific survival (P =7.542e-3) and progression-free survival (P =0.0578) (Figure 4D; Supplementary Table S2).

### 3.5. Alternative splicing analysis of CDCA5

**Figure 5:**
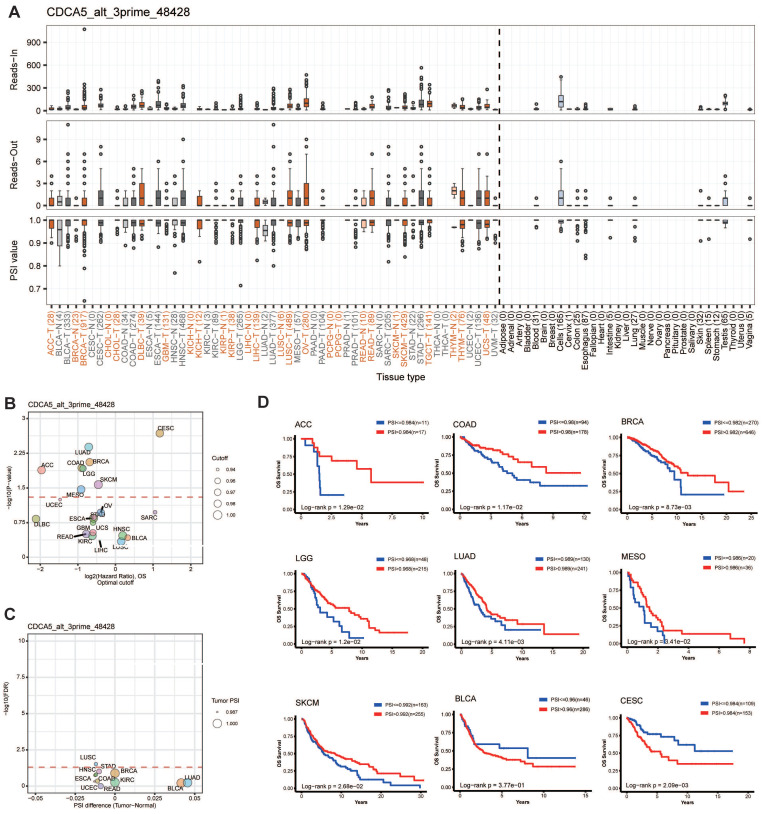
CDCA5 alternative splicing correlated to patient prognosis. (A) The splicing mode and the PSI of CDCA5_alt_3prime_48428 in pan-cancer. (B) Prognosis of CDCA5_alt_3prime_48428 event in 33 types of TCGA Cancers. (C) Difference in PSI values of the CDCA5_alt_3prime_48428 event between the tumor and normal tissues. (D) Kaplan-Meier curves of patients' OS and PFI prediction are plotted.

Alternative splicing (AS) is a frequent kind of post-transcriptional modification that generates diverse transcripts, proteins, and noncoding RNAs. Its dysregulation is common in malignancies and influences carcinogenesis 24. We analyzed the AS events on OncoSplicing, only one known clinical relevant CDCA5_alt_3prime_48428 event was identified. Figure 5A depicted the splicing mechanism and PSI of CDCA5_alt_3prime_48428 in pan-cancer; OV and STAD malignancies exhibit a higher PSI compared to normal samples. Figure 5C showed the difference in PSI values of the CDCA5_alt_3prime_48428 event between malignant and normal tissues, together with its prognosis in 33 types of TCGA Cancers (Figure 5B). High PSI predicted longer OS in ACC, COAD, BRCA, LGG, LUAD, MESO and SKCM. However, elevated PFI was found to be significantly associated with reduced overall survival in patients with BLCA and CESC, as illustrated in Figure 5D. The significance of controlled CDCA5 As events in the progression of cancer was indicated by these data.

### 3.6. The relationship between CDCA5 expression and immune infiltrating level

**Figure 6:**
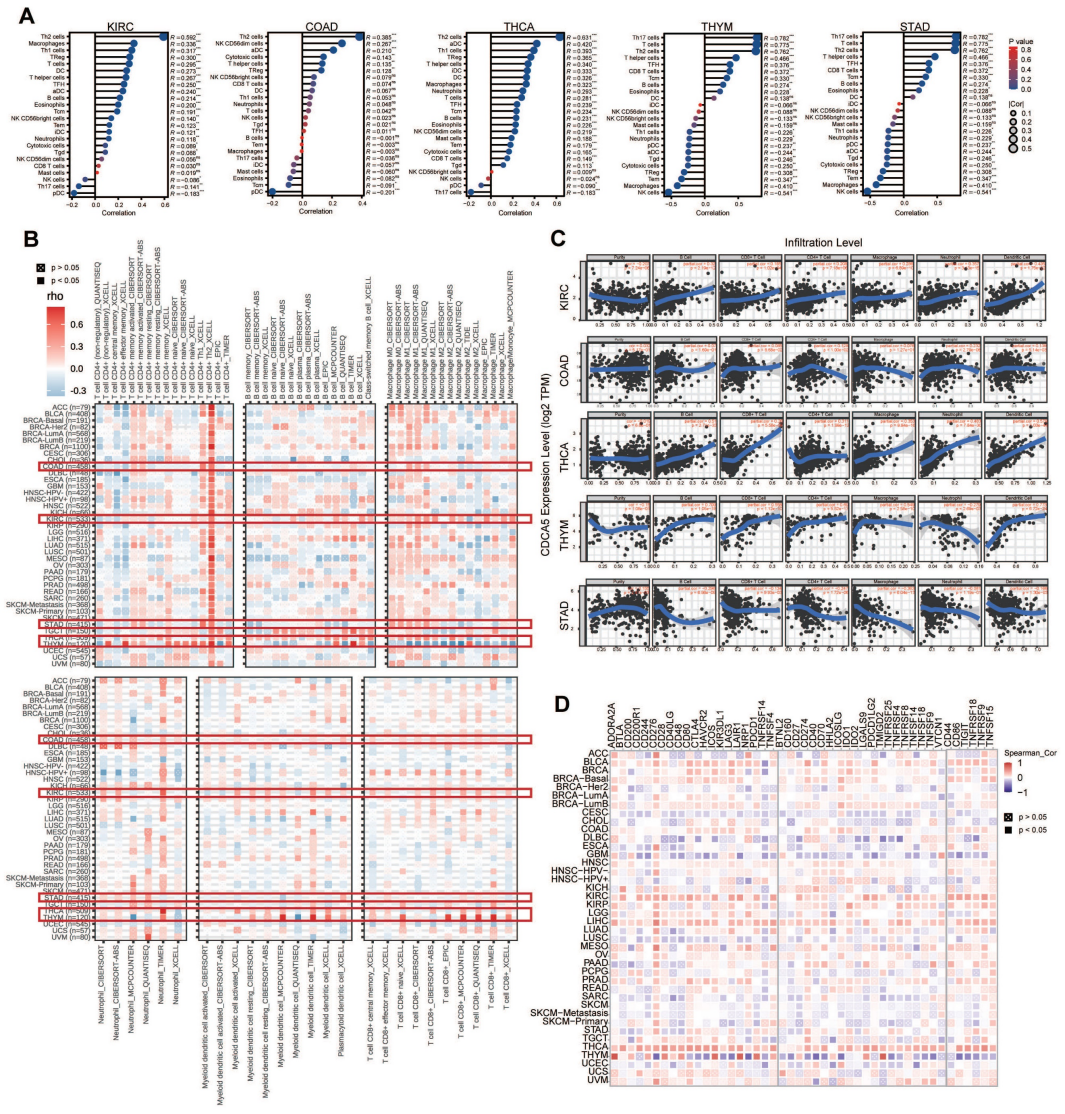
Correlation analysis between CDCA5 expression and immune microenvironment. (A) Lollipop chart comparing the degree of infiltration of the 24 immune cells in KIRC, COAD, THCA, THYM, and STAD. (B) Associations between CDCA5 expression and the degree of immune cell infiltration in different malignancies utilizing the infiltration scores of six immune cell types (B cell, CD4+ T cell, CD8+ T cell, neutrophil, macrophage, and dendritic cell) from the TIMER2 database and TCGA. (C) A strong connection between CDCA5 expression and the degree of immune cell infiltration in 5 tumors (KIRC, COAD, THCA, THYM, and STAD) using the infiltration scores of six immune cell types (B cell, CD4+ T cell, CD8+ T cell, neutrophil, macrophage, and dendritic cell) accessible in the TIMER database and obtained from TCGA. (D) The correlation between CDCA5 and immune checkpoints in different cancer types.

We examined the correlation between CDCA5 expression and immune cell infiltration levels in various types of cancer using TCGA data. We utilized R packages to compare the degree of immune cell infiltration and identified a significant correlation between immune cells and various cancers, including KIRC, COAD, STAD, THCA, and THYM (Figure 6A). Using the infiltration scores of six immune cell types (B cell, CD4+ T cell, CD8+ T cell, neutrophil, macrophage, and dendritic cell) accessible through the TIMER and TIMER2 databases, we further study the link between the six immune cells and these five tumors (Figure 6B). According to the attached linear regression plots from KIRC, COAD, and THCA, an increase in immune cell infiltration is associated with an increase in CDCA5 expression. Except for neutrophils, a rise in immune cell infiltration is associated with a rise in CDCA5 expression in THYM. In contrast, the relationship between CDCA5 expression and immune cell infiltration in STAD is negative (Figure 6C).

### 3.7. The relationship between immune checkpoints and CDCA5

Multiple genes are now closely linked to and acknowledged as components of immune response checkpoints. With the utilization of TIMER2, we were able to assess whether there exists a correlation between CDCA5 expression and the expression of checkpoint genes. First, in THYM, CDCA5 has positive relationships with ADORA2A, CD200, CD244, CD28, CD40LG, TNFSF4, CD27, HHLA2, ICOS, LAIR1, PDCD1, TM1GD2, and LGALS9, whereas it has negative relationships with CD276, NRP1, LAG3, CD40, ICOSLG, CD86, etc. Furthermore, a significant correlation was observed between CDCA5 and the majority of immune checkpoints in THCA. A moderately positive connection existed between CDCA5 and immune checkpoints in LIHC, KIRC, BLCA, and COAD. Moreover, CDCA5 exhibited a negative correlation with the majority of immune checkpoints in both CESC and GBM. It is noteworthy that a strong correlation (P<0.05) between CDCA5 and checkpoint gene expression was observed, which was found to be associated with immune gene CD276 in various types of cancer (Figure 6D).

### 3.8. The relationship between CDCA5 and TME

**Figure 7:**
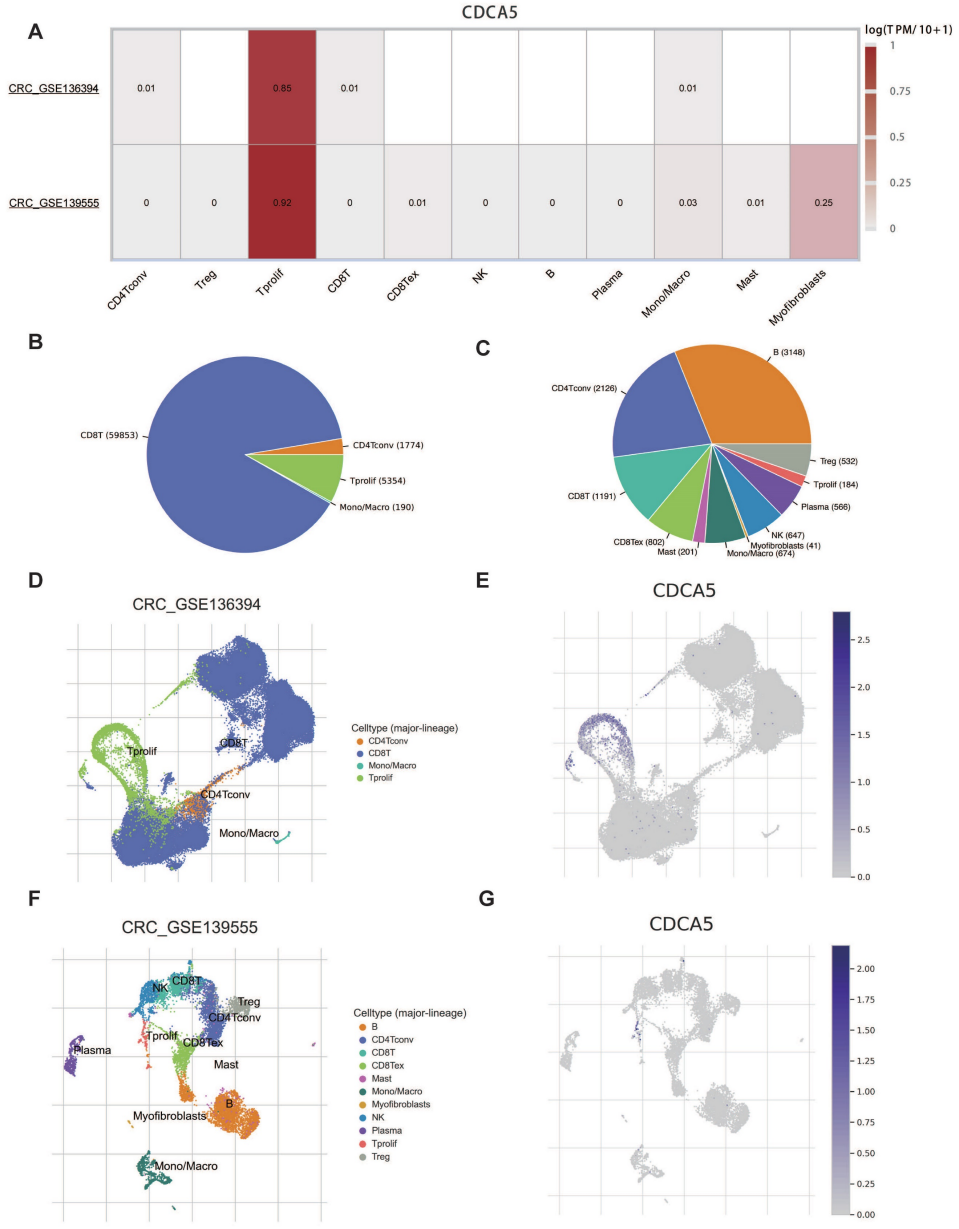
Correlation between CDCA5 and TME of colorectal cancer with TISCH database. (A) Correlation of CDCA5 with TME in CRC. (B, C) The annotation and distribution of the immune cell types in CRC GSE136394 and CRC GSE13955. The proportion of CDCA5 in different immune cell types in CRC GSE136394 dataset (D, E) and CRC GSE13955 dataset (F, G).

Given that the results of COAD were significant in multiple analyses, we focused on colorectal cancer (CRC) to learn more. Two datasets of colorectal cancer (CRC GSE136394 and CRC GSE139555) from TISCH were analyzed to investigate the correlation between CDCA5 expression and tumor microenvironment (TME). CDCA5 expression in CRC was concentrated in proliferating T cells, as illustrated in Figure 7A. Figure 7B and 7C depict the distribution and 12 categories of immune-related cells in the TME. It is worth noting that there was a positive correlation between CDCA5 expression and the extent of infiltrating T cell proliferation (Figure 7D-G). Consequently, our findings revealed that CDCA5 may influence the TME and that proliferating T cells could play a critical role in CRC.

### 3.9. Enrichment analysis of CDCA5

**Figure 8:**
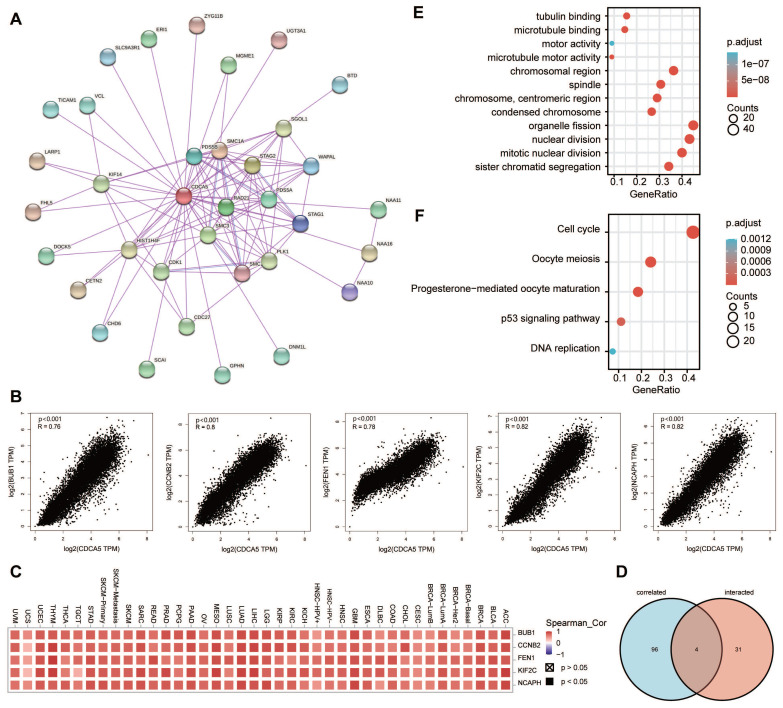
CDCA5-related gene enrichment analysis. (A) Using the STRING tool, we obtained the available experimentally determined CDCA5-binding proteins. (B) We retrieved the top 100 CDCA5-correlated genes in TCGA projects using the GEPIA2 method and evaluated the expression correlation between CDCA5 and chosen targeted genes, including BUB1, CCNB2, FEN1, KIF2C and NCAPH. (C) The matching heatmap data for each form of cancer is displayed. (D) We did an intersection analysis of the CDCA5-binding and associated genes. (E) On the basis of CDCA5-binding and interacting genes, GO analysis was conducted. (F) On the basis of CDCA5-binding and interacting genes, KEGG pathway analysis was conducted.

To further examine the biological function of the CDCA5 gene in carcinogenesis, we screened for proteins that bound to CDCA5 and genes whose expression was correlated with CDCA5, followed by pathway enrichment studies. The STRING database was used to identify 35 CDCA5-binding proteins supported by experimental data. The protein interaction network is illustrated in Figure 8A. We utilized the GEPIA2 method to aggregate all TCGA tumor expression data and found the top 100 genes whose expression was linked with CDCA5 expression. The expression level of CDCA5 is positively correlated with the genes BUB1 (R=0.76), CCNB2 (R=0.8), FEN1 (R=0.78), KIF2C (R=0.82) and NCAPH (R=0.82) as depicted in Figure 8B, with a significance level of P<0.001. In most of the designated cancer types, the matching heatmap data likewise revealed a positive connection between CDCA5 and the mentioned five genes (Figure 8C). An analysis of the overlap between the two aforementioned groups revealed four common members, namely PLK1, SGOL1, CDK1 and KIF14 (Figure 8D; Supplementary table S3).

We integrated the two datasets to conduct enrichment studies for KEGG and GO. The majority of these genes are associated to the cell cycle, according to GO enrichment analysis results, such as “nuclear division”, “sister chromatid segregation” and “mitotic nuclear division” (Figure 8E). The KEGG data indicates that “DNA replication” and “cell cycle” may have a role in the influence of CDCA5 on tumor pathogenesis (Figure 8F).

### 3.10. CDCA5 silencing decreases cell proliferation and enhances apoptosis

**Figure 9:**
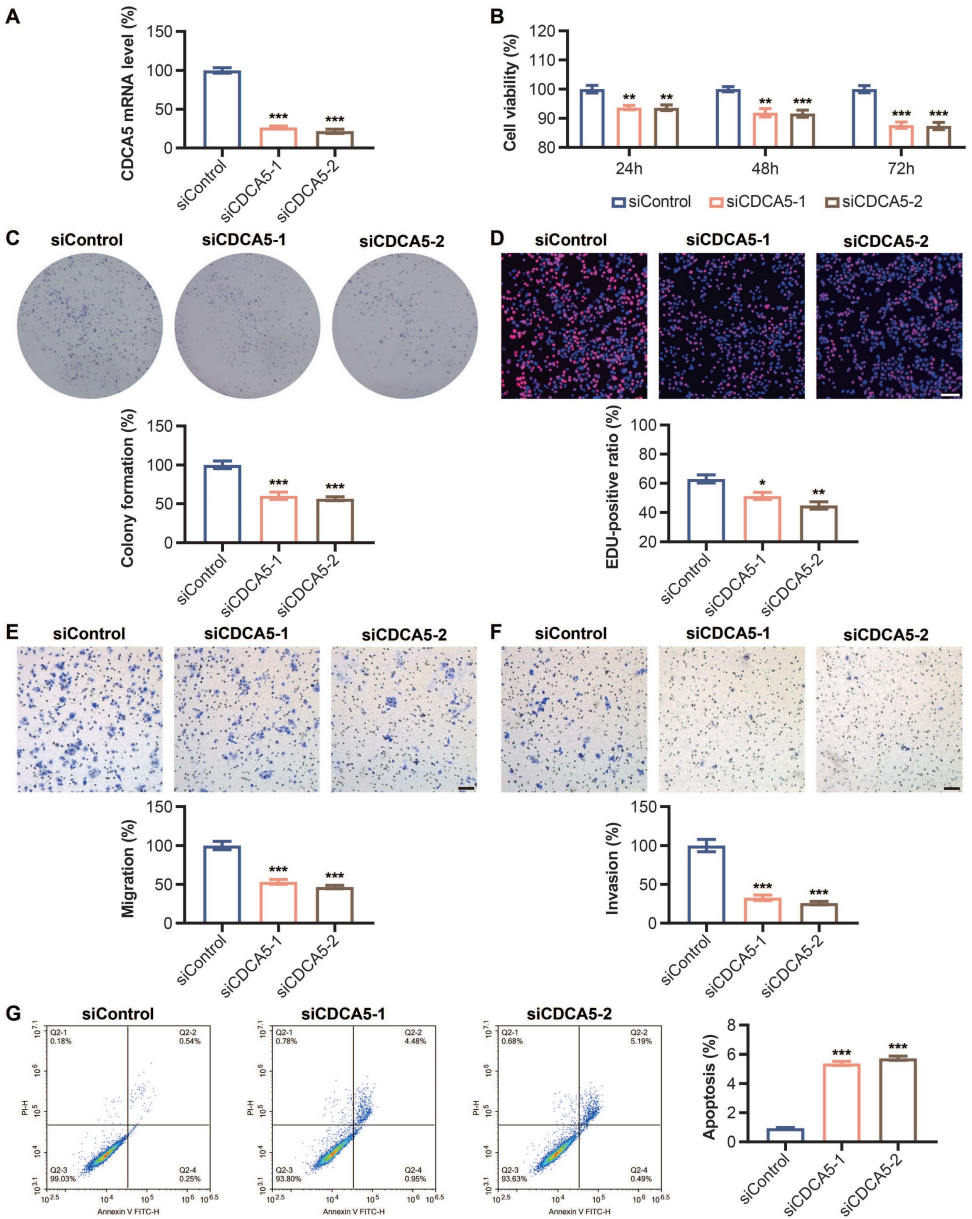
Effect of CDCA5 silencing on DLD1 cell proliferation, apoptosis, migration and clone formation abilities. (A) mRNA level of CDCA5 after Si-CDCA5 transfection. (B) The viability of DLD1 cells after Si-CDCA5 transfection. (C) Cell clone formation after CDCA5 knockdown. (D) Cell proliferation after CDCA5 knockdown. (E) CDCA5-siRNA reduced the migration of colon cancer cells. (F) CDCA5-siRNA reduced the invasion of colon cancer cells. (G) Cell apoptosis after CDCA5 knockdown. *p < 0.05, **p < 0.01, ***p < 0.001, ****p < 0.001.

In DLD1 cells transfected with CDCA5 siRNA, the mRNA expression level of CDCA5 was dramatically reduced compared to that of Si-Ctrl, showing that CDCA5 was successfully knocked down (Figure 9A). Significantly lower cell viability was seen following CDCA5 silencing (Figure 9B). Figure 9D demonstrates that the percentage of positive EdU-labeled signals decreased dramatically after CDCA5 expression was decreased. Flow cytometric analysis found an increase in early and overall apoptosis rates after CDCA5 downregulation (Figure 9G).

### 3.11. CDCA5 silencing inhibits cell migration and clone formation

After transfection with CDCA5 siRNA, the migration and invasion abilities of DLD1 cells were dramatically reduced compared to Si-Control. Figures 9E and 9F displayed the quantitative results of the migration experiment and invasion assay, respectively. In addition, as depicted in Figure 9C, CDCA5 knockdown impeded the capacity of DLD1 cells to generate clones.

## 4. Discussion

Cancer is a severe danger to human health and the leading cause of illness and mortality on a global scale 1. Recently, the focus of cancer research and therapy has shifted to the molecular and genetic levels with the advent of innovative treatments 25. The CDCA5 gene encodes the sororin-containing CDCA5 protein, which was initially discovered as a substrate of the anaphase-promoting (APC) complex and was shown to regulate sister chromatid cohesion 26, 27. Emerging papers have suggested a functional connection between CDCA5 and clinical pathologies, including cancer 7-10. The question of whether CDCA5 can have a role in the pathogenesis of a variety of cancers via shared molecular processes is unsolved.

Previous research has demonstrated that downregulation of CDCA5 suppresses several tumor growth 7-10. However, the expression of CDCA5 in 33 tumors has not been comprehensively studied. In our study, we discovered that CDCA5 is highly expressed in the majority of cancers and is extensively expressed in a range of organs. Studies have shown a correlation between decreased DNA methylation and an increase in mRNA expression 28. Gene expression analysis of CDCA5 reveals that CDCA5 promoter methylation levels, mRNA and protein expression levels were consistent in KIRC, COAD, HNSC, LUAD, and UCEC, and high CDCA5 expression was related with the development of cancer. In addition, increased CDCA5 expression is highly related to the advanced stage of cancer, indicating malignant development. Survival study also demonstrated a correlation between CDCA5 overexpression and poor OS and DFS. Accumulating evidence suggests that genetic alterations play a role in tumor growth and treatment response 29. To determine if CDCA5 mutations affect the clinical outcomes of cancer patients, we found that CDCA5 alterations may be a risk factor for esophageal adenocarcinoma patients. Former studies have demonstrated that alternative splicing increases transcriptome diversity, and nearly all human multiexon genes undergo this process 30. To further explore the changes at the gene expression level, we assess the expression level of CDCA5 in malignancies with respect to alternative splicing, and we compute a score based on the OS event and PSI difference, where OV and STAD samples had a higher PSI than normal samples. The analysis of splicing factor survival based on PSI values demonstrates that CDCA5 is significantly related with the overall survival of numerous cancers, including ACC, COAD, BLCA, etc. Knowing these helps answer questions about the pathophysiology of cancer and ultimately confirms CDCA5 as a potential therapy target.

Immune cells interact greatly with cancer cells and play a crucial influence on cancer migration and metastasis in several types of tumors 31, 32. The tumor microenvironment (TME) is crucial for the proliferation and development of tumor cells 33. Thus, we have shed light on the association between CDCA5 expression level and immune cell infiltration in certain cancers. Elevated CDCA5 expression is associated with an increase in immune cell infiltration in KIRC, COAD and THCA, which suggests that CDCA5 is closely related to tumor TME. It is noteworthy that the expression level of CDCA5 has a correlation with the immune infiltration level in THYM, except for neutrophil. Former studies demonstrated that neutrophils may assist in cancer metastasis promotion through different methods 34-36. Therefore, it may explain why CDCA5 overexpression was related with a good prognosis in THYM. Multiple studies have demonstrated that immune checkpoint genes are highly expressed in a diverse range of tumors and interact positively with tumor progression and poor prognosis 37. One of the most important novel cancer therapy techniques is immunotherapy and the most commonly used drugs in immunotherapy are those that target and suppress immune checkpoint pathways 38, 39. However, the relationship between CDCA5 expression and immune checkpoint has not been studied. Our study revealed a strong connection between CDCA5 and the immune gene CD276 in various cancer types. There is evidence to support the notion that the CD276, also referred to as B7 homolog 3 protein (B7-H3), is a recently discovered member of the B7 family of immunoregulatory proteins. It has garnered significant interest as a potential target for cancer immunotherapy due to its high expression in tumor tissues and low expression in normal tissues 40. Additionally, B7-H3 plays a role in shaping and developing TME. To date, several immunotherapy approaches utilizing B7-H3 have exhibited robust anticancer efficacy and satisfactory safety profiles in preclinical animal models 41. The results of our study hold promise for generating novel concepts within the realm of tumor immunotherapy, therefore fostering the advancement of inventive therapeutic strategies. The association between CDCA5 and immunological checkpoints in LIHC, KIRC, BLCA, COAD and THCA was positive, so it is not difficult to comprehend why CDCA5 overexpression was related with a bad prognosis in these four cancers. Then we focused on colorectal cancer to learn more about the relationship between CDCA5 expression and TME. The TISCH study proved that CDCA5 was moderately expressed in immune cells and had a strong association with proliferating T cells, indicating that CDCA5 was implicated in the immune regulatory network by enhancing immune activation in colorectal cancer. Collectively, our research illuminates the underlying role of CDCA5 in cancer immunity.

Integrating information on the CDCA-binding proteins and the genes related with CDCA5 expression across the various cancer types, the findings of enrichment analysis showed the potential function of CDCA5 protein. Our investigation revealed that the CDCA5 expression level is positively connected with the BUB1, CCNB2, FEN1, KIF2C, and NCAPH genes, all of which produce proteins involved in cell cycle regulation and DNA repair 42-46. Based on the GO and KEGG pathway analyses, we found that CDCA5 may be involved in the signal control of the p53 pathway via altering genes involved in the cell cycle, which was consistent with the findings of prior research 47. Given that the COAD results were significant in multiple analyses and that the relationship between COAD and CDCA5 had not been investigated, we concentrated on colon cancer to verify the relationship between CDCD5 and tumors. The results of cell experiments showed that CDCA5 knockdown inhibits the growth of DLD1 cells and the capacity of DLD1 cells to generate clones, but increases early and overall apoptosis rates. The DNA replication activity of the cells was diminished and their potential to proliferate was decreased. The migration and invasion abilities of DLD1 cells were also dramatically reduced after CDCA5 silencing. Our findings demonstrated that CDCA5 promoted cancer cell growth and inhibited apoptosis in vitro, which were consistent to the bioinformatic analyses.

## 5. Conclusion

Our study was the first to do a thorough pan-cancer investigation of CDCA5, and the results demonstrated a significant link between CDCA5 expression and clinical prognosis, DNA methylation, tumor mutation, alternative splicing, immune infiltration, immune checkpoints, tumor microenvironment and protein interaction network, which contributes to the knowledge of the role of CDCA5 in carcinogenesis through the perspective of clinical tumor samples.

## Supplementary Material

Supplementary figure and tables.
