# Supplementary material for: Integrated Multi-omics Analyses Identify CDCA5 as a Novel Biomarker Associated with Alternative Splicing, Tumor Microenvironment, and Cell Proliferation in Colon Cancer Via Pan-cancer Analysis
Source: J Cancer. 2024 Jan 1;15(3):825–40. doi: 10.7150/jca.91082 (PMC10777042; doi:10.7150/jca.91082)
Supplement: Supplementary file 1 — Supplementary figure and tables. [file jcav15p0825s1.pdf]

Tables:

Supplementary table 1: The alternation information of CDCA5.

Study of Origin	Sample ID	Cancer Type	Cancer Type Detailed
Glioblastoma Multiforme (TCGA, PanCancer Atlas)	TCGA-06-5416-01	Glioblastoma	Glioblastoma Multiforme
Glioblastoma Multiforme (TCGA, PanCancer Atlas)	TCGA-14-1458-01	Glioblastoma	Glioblastoma Multiforme
Lung Squamous Cell Carcinoma (TCGA, PanCancer Atlas)	TCGA-18-4721-01	Non-Small Cell Lung Cancer	Lung Squamous Cell Carcinoma
Lung Squamous Cell Carcinoma (TCGA, PanCancer Atlas)	TCGA-22-5491-01	Non-Small Cell Lung Cancer	Lung Squamous Cell Carcinoma
Lung Squamous Cell Carcinoma (TCGA, PanCancer Atlas)	TCGA-56-8626-01	Non-Small Cell Lung Cancer	Lung Squamous Cell Carcinoma
Lung Squamous Cell Carcinoma (TCGA, PanCancer Atlas)	TCGA-77-6845-01	Non-Small Cell Lung Cancer	Lung Squamous Cell Carcinoma
Lung Squamous Cell Carcinoma (TCGA, PanCancer Atlas)	TCGA-MF-A522-01	Non-Small Cell Lung Cancer	Lung Squamous Cell Carcinoma
Bladder Urothelial Carcinoma (TCGA, PanCancer Atlas)	TCGA-DK-A3IT-01	Bladder Cancer	Bladder Urothelial Carcinoma
Bladder Urothelial Carcinoma (TCGA, PanCancer Atlas)	TCGA-BT-A20W-01	Bladder Cancer	Bladder Urothelial Carcinoma
Bladder Urothelial Carcinoma (TCGA, PanCancer Atlas)	TCGA-E5-A4U1-01	Bladder Cancer	Bladder Urothelial Carcinoma
Bladder Urothelial Carcinoma (TCGA, PanCancer Atlas)	TCGA-MV-A51V-01	Bladder Cancer	Bladder Urothelial Carcinoma
Liver Hepatocellular Carcinoma (TCGA, PanCancer Atlas)	TCGA-DD-AADB-01	Hepatobiliary Cancer	Hepatocellular Carcinoma
Prostate Adenocarcinoma (TCGA, PanCancer Atlas)	TCGA-XK-AAIW-01	Prostate Cancer	Prostate Adenocarcinoma

Uterine Corpus Endometrial Carcinoma (TCGA, PanCancer Atlas)	TCGA-D1-A167-01	Endometrial Cancer	Uterine Endometrioid Carcinoma
Uterine Corpus Endometrial Carcinoma (TCGA, PanCancer Atlas)	TCGA-A5-A0G2-01	Endometrial Cancer	Uterine Serous Carcinoma/Uterine Papillary Serous Carcinoma
Uterine Corpus Endometrial Carcinoma (TCGA, PanCancer Atlas)	TCGA-AP-A051-01	Endometrial Cancer	Uterine Endometrioid Carcinoma
Uterine Corpus Endometrial Carcinoma (TCGA, PanCancer Atlas)	TCGA-AX-A0J1-01	Endometrial Cancer	Uterine Endometrioid Carcinoma
Uterine Corpus Endometrial Carcinoma (TCGA, PanCancer Atlas)	TCGA-AP-A1DV-01	Endometrial Cancer	Uterine Endometrioid Carcinoma
Uterine Corpus Endometrial Carcinoma (TCGA, PanCancer Atlas)	TCGA-AX-A1C9-01	Endometrial Cancer	Uterine Endometrioid Carcinoma
Uterine Corpus Endometrial Carcinoma (TCGA, PanCancer Atlas)	TCGA-BG-A222-01	Endometrial Cancer	Uterine Endometrioid Carcinoma
Lung Adenocarcinoma (TCGA, PanCancer Atlas)	TCGA-55-7727-01	Non-Small Cell Lung Cancer	Lung Adenocarcinoma
Esophageal Adenocarcinoma (TCGA, PanCancer Atlas)	TCGA-L5-A8NN-01	Esophagogastric Cancer	Esophageal Adenocarcinoma
Skin Cutaneous Melanoma (TCGA, PanCancer Atlas)	TCGA-EE-A2GT-06	Melanoma	Cutaneous Melanoma
Skin Cutaneous Melanoma (TCGA, PanCancer Atlas)	TCGA-FR-A726-01	Melanoma	Cutaneous Melanoma
Skin Cutaneous Melanoma (TCGA, PanCancer Atlas)	TCGA-ER-A193-06	Melanoma	Cutaneous Melanoma
Skin Cutaneous Melanoma (TCGA, PanCancer Atlas)	TCGA-ER-A193-06	Melanoma	Cutaneous Melanoma
Skin Cutaneous Melanoma (TCGA, PanCancer Atlas)	TCGA-EE-A183-06	Melanoma	Cutaneous Melanoma
Skin Cutaneous Melanoma (TCGA, PanCancer Atlas)	TCGA-ER-A19P-0	Melanoma	Cutaneous Melanoma

	6		
Skin Cutaneous Melanoma (TCGA, PanCancer Atlas)	TCGA-HR-A2OG-06	Melanoma	Cutaneous Melanoma
Stomach Adenocarcinoma (TCGA, PanCancer Atlas)	TCGA-BR-4361-01	Esophagogastric Cancer	Stomach Adenocarcinoma
Breast Invasive Carcinoma (TCGA, PanCancer Atlas)	TCGA-AO-A0JB-01	Breast Cancer	Breast Invasive Ductal Carcinoma
Pancreatic Adenocarcinoma (TCGA, PanCancer Atlas)	TCGA-IB-7651-01	Pancreatic Cancer	Pancreatic Adenocarcinoma
Colorectal Adenocarcinoma (TCGA, PanCancer Atlas)	TCGA-AG-A015-01	Colorectal Cancer	Rectal Adenocarcinoma
Head and Neck Squamous Cell Carcinoma (TCGA, PanCancer Atlas)	TCGA-CR-5248-01	Head and Neck Cancer	Head and Neck Squamous Cell Carcinoma
Head and Neck Squamous Cell Carcinoma (TCGA, PanCancer Atlas)	TCGA-CQ-5331-01	Head and Neck Cancer	Head and Neck Squamous Cell Carcinoma
Head and Neck Squamous Cell Carcinoma (TCGA, PanCancer Atlas)	TCGA-CQ-A4CD-01	Head and Neck Cancer	Head and Neck Squamous Cell Carcinoma
Cervical Squamous Cell Carcinoma (TCGA, PanCancer Atlas)	TCGA-Q1-A73P-01	Cervical Cancer	Endocervical Adenocarcinoma
Cervical Squamous Cell Carcinoma (TCGA, PanCancer Atlas)	TCGA-Q1-A73O-01	Cervical Cancer	Cervical Squamous Cell Carcinoma
Cervical Squamous Cell Carcinoma (TCGA, PanCancer Atlas)	TCGA-Q1-A73O-01	Cervical Cancer	Cervical Squamous Cell Carcinoma
Cervical Squamous Cell Carcinoma (TCGA, PanCancer Atlas)	TCGA-2W-A8YY-01	Cervical Cancer	Cervical Squamous Cell Carcinoma

Supplementary table 2: Survival status of EAC patients with altered CDCA5

Survival Type	Number of Patients	# in Altered group	# in Unaltered group	Median months survival in Altered group (95% CI)	Median months survival in Unaltered group (95% CI)	p-Value	q-Value
Disea	180	6	174	6.35 (4.31 - NA)	46.09 (31.27 - NA)	0.0	0.0

se-specific						07542	302
Progression Free	182	6	176	6.35 (3.22 - NA)	18.81 (15.68 - 28.11)	0.0578	0.106
Overall	182	6	176	6.35 (4.31 - NA)	26.33 (22.39 - 46.09)	0.0797	0.106
Disease Free	87	1	86	NA	NA	0.616	0.616

Supplementary table 3: An intersection analysis of the CDCA5-binding and associated genes.

Only correlated	Only interacted	correlated AND interacted
NCAPH	BTD	PLK1
KIF2C	CDC27	SGOL1
CCNB2	CDCA5	CDK1
KIFC1	CETN2	KIF14
BIRC5	CHD6	
FEN1	DNM1L	
TPX2	DOCK5	
KIF4A	ERI1	
CCNB1	FHL5	
BUB1	GPHN	
NCAPG	HIST1H4F	
RACGAP1	LARP1	
DLGAP5	MGME1	
CENPA	NAA10	
HJURP	NAA11	
CCNA2	NAA16	
SKA1	PDS5A	
POLA2	PDS5B	
CHEK1	RAD21	
KIF11	SCAI	
AURKB	SLC9A3R1	
OIP5	SMC1A	
EXO1	SMC1B	
RAD51	SMC3	
GTSE1	STAG1	
KIF23	STAG2	

CCNF	TICAM1	
ZWINT	UGT3A1	
TROAP	VCL	
RAD54L	WAPAL	
PLK4	ZYG11B	
PRC1		
AUNIP		
TTK		
SPC25		
KPNA2		
MELK		
NUSAP1		
CDCA3		
CENPO		
MAD2L1		
CDC45		
GINS1		
TRAIP		
MCM10		
TIMELESS		
ORC6		
CKAP2L		
CDC25C		
POC1A		
CDC20		
MKI67		
LMNB2		
C16orf59		
C17orf53		
FOXM1		
FANCI		
MCM6		
CEP55		
ALYREF		
CENPI		
CDCA8		
KIF15		
UBE2T		
KIF20A		
MCM2		
MTFR2		
UBE2S		
DSCC1		

KIF18B		
UBE2C		
AURKA		
NDC80		
CENPF		
KIF18A		
LMNB1		
NCAPG2		
MND1		
RRM2		
CENPH		
EZH2		
KIAA1524		
GSG2		
CDT1		
CKS1B		
INCENP		
ASPM		
CDCA4		
CHAF1A		
CDKN3		
NUF2		
SHCBP1		
LRR1		
DDIAS		
ORC1		
RAN		

Legend:
Supplementary table 1: cBioPortal is used to find the alternation information of CDCA5.
Supplementary table 2: Survival status of EAC patients with altered CDCA5 are studied from cBioPortal.
Supplementary table 3: The CDCA5-binding and associated genes were analyzed jointly to find the Intersection genes.

Figure S1:

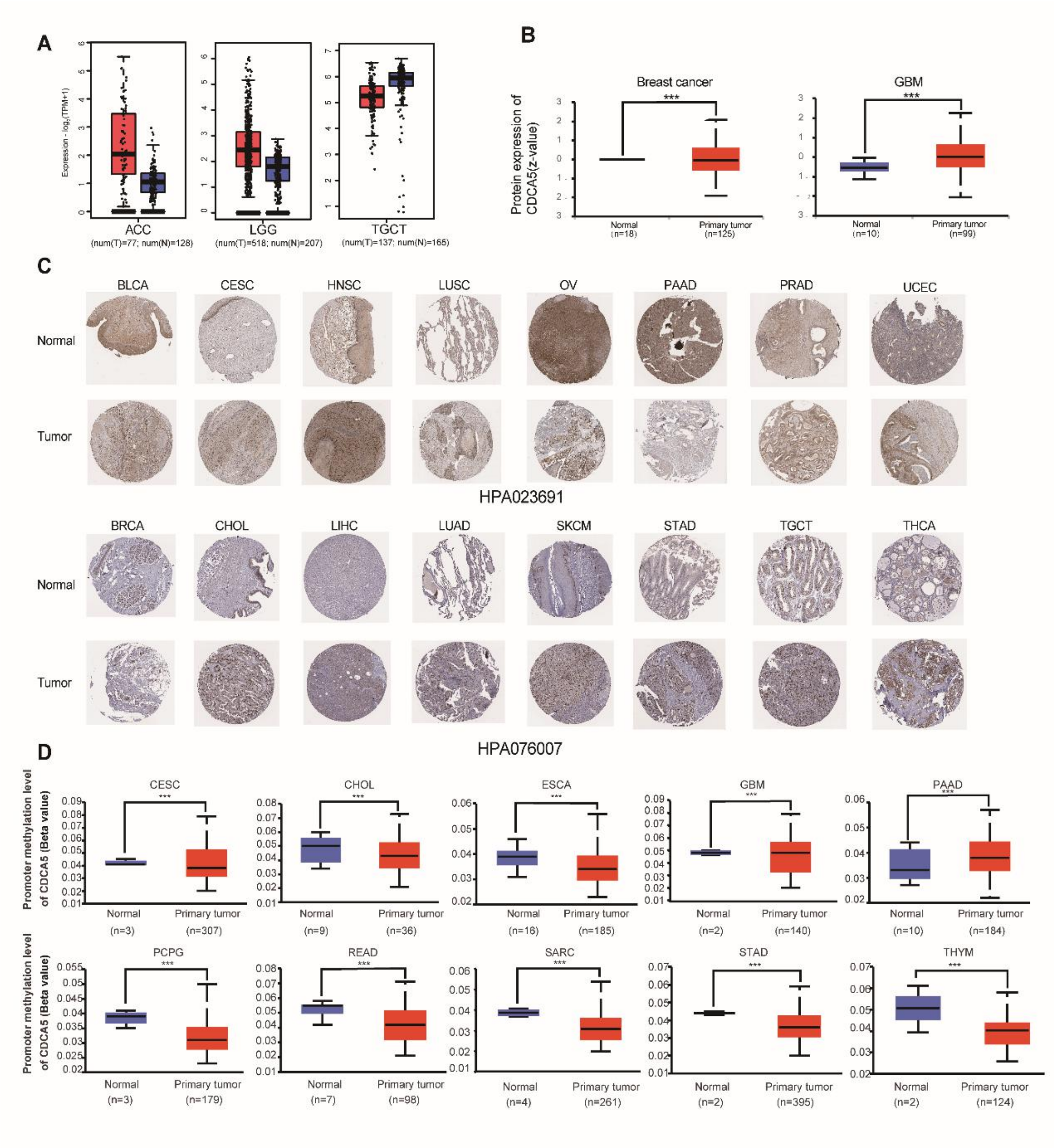

Legend:

Figure S1. The expression status of CDCA5 gene in various tumors. (A) As controls for the ACC, LGG and TGCT types in the TCGA project, the matching normal tissues from the GTEx database were included. There is no difference between normal tissue and primary tumor in these 3 cancers. (B) The expression level of CDCA5 total protein between normal tissue and primary tissue of breast cancer and GBM was compared based on the data from CPTAC. (C) IHC results obtained from the HPA database, display that expression of CDCA5 protein varies between normal tissues and tumors in distinct cancer types. Antibody HPA023691: BLCA, CESC, HNSC, LUSC, OV, PAAD, PRAD, and UCEC. Antibody HPA076007: BRCA, CHOL, LIHC, LUAD, SKCM, STAD, TGCT, and THCA. (D) The promoter methylation levels of CDCA5 across 10 types of tumors were analyzed in TCGA project using UALCAN.
